# Range extension of *Myotis midastactus*​ (Chiroptera, Vespertilionidae) to Paraguay

**DOI:** 10.3897/BDJ.3.e5708

**Published:** 2015-08-18

**Authors:** Ricardo Moratelli, Liu Idárraga, Don Ellis Wilson

**Affiliations:** ‡ Fiocruz Mata Atlântica, Fundação Oswaldo Cruz, Rio de Janeiro, Brazil; § Division of Mammals, National Museum of Natural History, Smithsonian Institution, Washington, DC, United States of America; | División de Mastozoología, Museo Argentino de Ciencias Naturales “Bernardino Rivadavia”, Ciudad Autónoma de Buenos Aires, Argentina

**Keywords:** *Myotis guaycuru*, *Myotis midastactus*, *Myotis simus*, South America, taxonomy

## Abstract

**Background:**

*Myotis midastactus* Moratelli and Wilson, 2014 (Vespertilionidae, Myotinae) was described from the *Myotis simus* Thomas, 1901 complex based on collections from the Bolivian Savannah.

**New information:**

Four vouchers previously assigned to *M. simus* from the Alto Chaco in Paraguay (West of the Paraguay River) are reassigned here to *M. midastactus*. These specimens extend the geographic distribution of *M. midastactus* 1200 km southward, and constitute the first evidence of the species in the country. Based on other material from the Brazilian Pantanal and Cerrado, Central Paraguay and north-eastern Argentina, we also discuss the identity of *simus*-like populations south of the Amazon Basin. The status of these populations is still unclear, but the little evidence we have at hand indicates that these populations may represent another taxon—*M. guaycuru* Proença, 1943; whereas *M. simus* seems to be restricted to the Amazon basin. This hypothesis is still very speculative and requires further investigation. With the assignment of material from Alto Chaco to *M. midastactus*, seven species of *Myotis* are confirmed for Paraguay: *M. albescens*, *M. lavali*, *M. levis*, *M. midastactus*, *M. nigricans*, *M. riparius*, and *M. ruber*.

## Introduction

*Myotis simus* was described by Thomas (1901) based on one specimen from Río Ucayali, Loreto, Peruvian Amazon. The species was known from tropical South American lowlands, with records east of the Andes in Colombia, Ecuador, Peru, Bolivia, Paraguay, northern and mid-western Brazil, and north-eastern Argentina (Wilson 2008, Moratelli et al. 2011b, Moratelli 2012). Previous records of *M. simus* from southern Brazil (Wallauer et al. 2000, Peracchi et al. 2006, Bianconi and Pedro 2007) and from west of the Ecuadorian Andes (Carrera et al. 2010) seem to be misidentifications of *M. levis* (I. Geoffroy, 1824) and *M. riparius* Handley, 1960, respectively (Cherem et al. 2004, Moratelli et al. 2011b, Moratelli et al. 2013).<br/>

Based on morphological and morphometric evidence (Moratelli et al. 2011a), Bolivian savannah populations were reassigned to a new species—*Myotis midastactus* Moratelli and Wilson, 2014; and populations from the Amazon lowlands in Colombia, Ecuador, Peru, Bolivia, and northern Brazil were retained as *M. simus* (see Moratelli and Wilson 2014). Populations from outside of the Amazon Basin, including those from the Brazilian Pantanal and Cerrado, Paraguay, and north-eastern Argentina, are represented by few specimens in collections. We examined four from west of the Paraguay River, Paraguay, and concluded they represent *M. midastactus*. A few others we examined from mid-western Brazil and north-eastern Argentina might represent neither *M. midastactus* nor *M. simus*. These specimens partially fit in the descriptions of *M. simus* provided by Thomas (1901) and LaVal (1973), but they differ slightly in the development of skull crests. In addition, a distribution modelling for *M. simus* (sensu LaVal 1973 and Wilson 2008) has supported the disjunctive distribution proposed by Wilson (2008), where Amazon Basin populations are isolated from southern South American populations by unsuitable habitats (Moratelli et al. 2011b). This disjunctive distribution was strengthened after the assignment of Bolivian savannah populations to *M. midastactus*. Based on this evidence, the taxonomic status of the southernmost populations of *M. simus* is pending further evaluation (Moratelli and Wilson 2014).<br/>

In this report we extend the geographic distribution of *M. midastactus* from the Bolivian savannah to west of the Paraguay River, Paraguay. We also discuss the status of populations of *M. simus* out of the Amazon Basin (herein referred as *simus*-like).

## Material and methods

Four pregnant females deposited as vouchers at the Museum of Vertebrate Zoology (MVZ), Berkeley, USA, constitute the first records of *M. midastactus* from Paraguay (MVZ 144481–144484). These vouchers were collected by P. Myers on 22 October 1972, 230 km NW from Villa Hayes (by road), Presidente Hayes, Paraguay (23°23′ S, 58°46′ W). Geographical coordinates for these specimens were obtained from Gardner's gazetteer of marginal localities (Gardner 2008). Identifications were based on the diagnostic characters reported by Moratelli and Wilson (2014).

**Figure 1.**
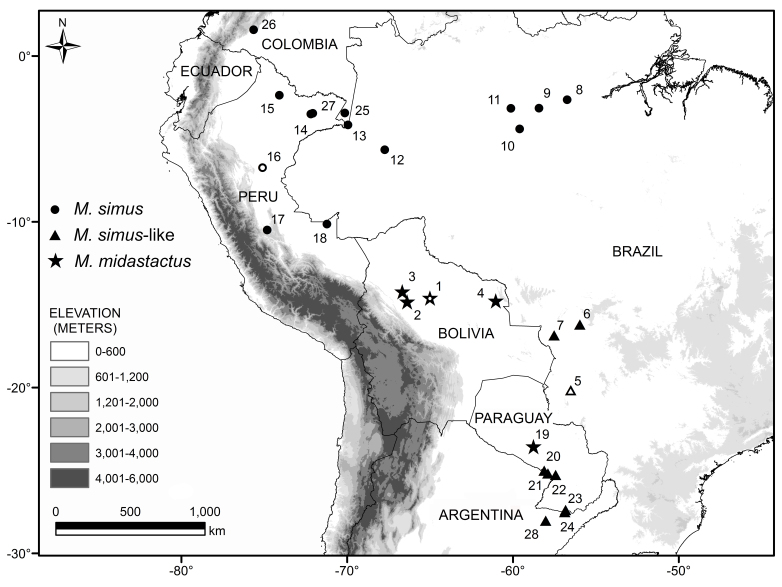
Map of part of South America illustrating localities for *Myotis midastactus* (stars), *M. simus* (circles), *M. simus*-like and other undetermined specimens (both represented by triangles). Type localities of *M. midastactus* (loc 1), *M. guaycuru* (loc 5), and *M. simus* (loc 16) are represented by symbols with white marks in the centre. See Supplementary material for localities and their geographical coordinates.

Qualitative and quantitative traits from the external and skull morphology were used to compare specimens representing *M. midastactus* from Paraguay with others representing *M. midastactus* (N = 33; type series) from Bolivia; *M. simus* (N = 79) from the Amazon Basin (northern Brazil, Colombia, Ecuador, and Peru); and *simus*-like (N = 8) from mid-western Brazil and northern Argentina (Figure 1). Sub-adults and adults were used in the qualitative analyses, but quantitative data was retrieved from adults only. These specimens and the geographical coordinates of their localities are listed in the supplementary material. We also compared *M. midastactus* from Paraguay with representatives of other species confirmed for the country except *M. levis*, which include: *M. albescens* (N = 161), *M. lavali* (N = 4), *M. nigricans* (N = 103), *M. riparius* (N = 10), and *M. ruber* (N = 5). *Myotis levis* is represented in Paraguay by a single record (Stevens et al. 2010), and characters used in the comparison were retrieved from Stevens et al. (2010).

Measurements in this report are either in millimetres (mm) or grams ([g] body mass) and are from adults. The total length (TL), tail, hind foot (HF), ear, and the body mass (BM) were recorded from skin labels. Other dimensions include: the forearm length (FA), third metacarpal length (3ML), length of the dorsal hair (LDH), length of the ventral hair (LVH), greatest length of skull, including incisors (GLS), condylocanine length (CCL), condylobasal length (CBL), condylo-incisive length (CIL), basal length (BAL), zygomatic breadth (ZB), mastoid breadth (MAB), braincase breadth (BCB), interorbital breadth (IOB), postorbital breadth (POB), breadth across canines (BAC), breadth across molars (BAM), maxillary toothrow length (MTL), molariform toothrow length (M1–3), mandibular length (MAL), and mandibular toothrow length (MAN). These measurements are defined in Moratelli et al. (2013). Skull measurements were taken under binocular dissection microscopes with low magnification (usually 6×). Dimensions were taken using digital callipers accurate to 0.02 mm. They were recorded and analysed to the nearest 0.01 mm, but values were rounded off to 0.1 mm throughout the text because this is the smallest unit that allows accurate repeatability with callipers (Voss et al. 2013). Descriptive statistics (mean and range) were calculated for all dimensions. Capitalized colour nomenclature follows Ridgway (1912).

## Results

### *Myotis midastactus*

**Figure 2.**
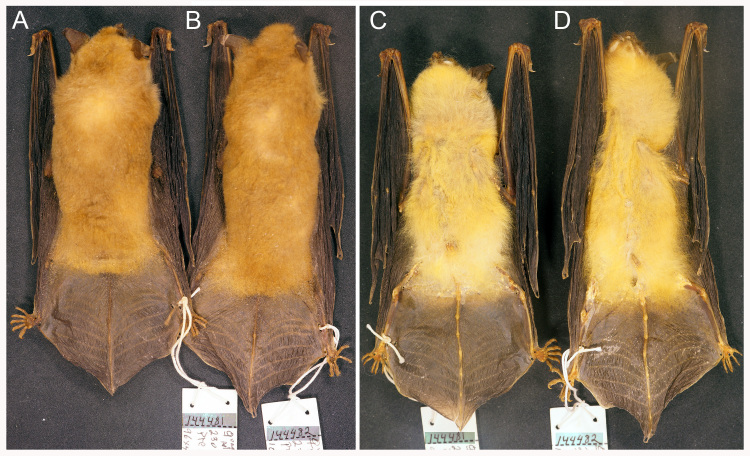
Dorsal (A, B) and ventral (C, D) pelage colours of *Myotis midastactus* (MVZ 144481, 144482) from Paraguay.

The four Paraguayan vouchers (MVZ 144481–144484) from Presidente Hayes (Fig. 1​, loc. 19), have the set of diagnostic character of *M. midastactus* provided by Moratelli and Wilson (2014) and fit their description of the especies. They have the woolly, extremely short (LDH 4–5 mm, LVH 3–5 mm), golden-yellow fur typical of the species (Fig. 2; Table 1​); plagiopatagium attached to the foot by a narrow band of membrane (≤ 1.5 mm); and mastoid breadth equal or larger than 7.8 mm (7.8–8.1 mm). The zygomatic breadth, which is part of the set of diagnostic characters, is not reported here because the arches are broken in these vouchers.

Like most other *M. midastactus*, the Paraguayan vouchers (MVZ 144481–144484) average larger than *M. simus* (Table 1), have mastoid processes more laterally projected, and paler and brighter dorsal and ventral fur colours. See Moratelli and Wilson (2014) for pictures comparing the pelage colours of *M. midastactus* and *M. simus*. In comparison with other species reported from Paraguay, they differ from *M. albescens*, *M. lavali*, *M. levis* and *M. nigricans* by the woolly fur (silky in *albescens*, *levis* and *nigricans*), and sagittal crest present and low (usually absent in *albescens*, *levis* and *nigricans*). In addition, they differ from *M. albescens* and *M. levis* by the absence of a fringe of hairs along the trailing edge of uropatagium. On the other hand, *M. lavali* has a yellowish general appearance but the dorsal fur is silky, longer, paler and strongly bicolored with blackish or dark-brownish basis in *M. lavali* (unicolor in *midastactus*). See pictures of the pelage colour of *M. lavali* in Moratelli et al. (2011c) and Moratelli and Wilson (2013). *Myotis midastactus* from Paraguay (MVZ 144481–144484) differ from *M. riparius* and *M. ruber* by the paler colour (reddish-brown or brownish in *riparius*, and reddish in *ruber*), shorter fur (ca. 7 mm in *riparius* and *ruber*), and plagiopatagium attached to the foot by a narrow band of membrane (plagiopatagium broadly attached to the foot in *riparius* and *ruber*).

### *Myotis simus*-like

Four specimens from three different localities in Argentina were tentatively assigned to *M. simus*-like. Two are from Formosa (CML 4680, MACN 20901 [Fig. 1, loc. 20]), and two are from Corrientes (MACN 18033 [Fig. 1, loc. 24]; MACN 20914 [Fig. 1, loc. 28]). CML 4680 and MACN 18033 are skin-only, and MACN 20901 and 20914 are composed of both skin and skull. They comprise all material available for “*M. simus*” in the main Argentinian collections.

These specimens match *M. simus* from the Amazon Basin in fur texture and length, and zygomatic breadth (Table 1). However, they lack sagittal and lambdoidal crests, and the dorsal fur colour is close to Buffy Brown or Dresden Brown, while those from the Amazon Basin generally have medium to high crests (Moratelli et al. 2011a​:50, table 4), with a dorsal fur colour ranging from Tawny, Russet and Cinnamon-brown. These Argentinian specimens are very close to the holotype of *M. guaycuru* in the skull morphology; and close to specimens from mid-western Brazil (MZUSP 13815, MN 71451, 71458) in fur colour, texture, and length.

### Other Paraguayan specimens

A few specimens in the *simus*-group from Paraguay are pending identification. They are from localities east of the Paraguay River (Fig. 1, locs 22, 23), reported by López-González et al. (2001), and López-González (2005). One is deposited in the Museum of Zoology, University of Michigan, Ann Arbor (UMMZ 125731), and the other possibly at the Museum of Texas Tech University (TK 60803 [museum abbreviation for the tissue collection]), but we did not locate the latter. Because López-González et al. (2001) and López-González (2005) provided descriptions and measurements of *M. midastactus* (MVZ 144481–144484) combined with the other two undetermined vouchers deposited at UMMZ and Texas Tech, we cannot make an attempt to assign them to either *M. midastactus* or *M. simus*. Nevertheless, both localities are geographically closer, and in a similar habitat, to the Argentinian localities of the specimens here reported as *simus-* like than the locality where we are reporting *M. midastactus*.

## Discussion

### *Myotis midastactus*

The Paraguayan vouchers we identified here as *M. midastactus* (MVZ 144481–144484) were previously identified as *M. simus* by Myers and Wetzel (1979) and Myers and Wetzel (1983), and part of them (MVZ 144483, 144484) were reidentified as *M. ruber* by Baud and Menu (1993). These vouchers extend the geographic distribution of *M. midastactus* in ca. 1200 km southward, and constitute the first records of the species for Paraguay. They enlarge the species distribution from the Beni Savannah (Fig. 1, locs 1–3) and Cerrado ecoregion (Fig. 1​, loc. 4) to the north of the Humid Chaco ecoregion (Fig. 1, loc. 19; ecoregion nomenclature following Olson et al. 2001). All records for this species are associated with flooded habitats (see Kingsbury 2010, Rumiz and Sainz 2002, López-González 2004).

*Myotis midastactus* was described from a Bolivian habitat that harbours a few endemic birds and small mammals (Olson et al. 2001, Emmons and Patton 2005) — e.g., *Hylaemys acritus* (Emmons and Patton, 2005) (Sigmodontinae, Oryzomyini [see Emmons and Patton 2005]) and *Ara glaucogularis* Dabbene, 1921 (Psittaciformes, Psittacidae [see Kingsbury 2010]). However, due to the wide distribution of other Neotropical congeners (see Moratelli et al. 2013), *M. midastactus* was expected to occur in other similar habitats. Based on preliminary and unpublished results, we have seen that several closely related species of Neotropical *Myotis* tend to conserve niche characteristics after speciation. Due to its morphological resemblance to *M. simus*, we speculate that *M. midastactus* is restricted to the tropical lowlands of Bolivia and Paraguay, although more empirical data is necessary to accurately define the geographic limits of this species. With the assignement of this material to *M. midastactus*, seven species of *Myotis* are confirmed for Paraguay: *M. albescens*, *M. lavali*, *M. levis*, *M. midastactus*, *M. nigricans*, *M. riparius*, and *M. ruber*. Among them, *M. albescens*, *M. lavali*, *M. levis*, and *M. nigricans* are in the *albescens*-group; and *M. midastactus*, *M. riparius*, and *M. ruber* are in the *ruber*-group (see Moratelli et al. 2013​).

### *Myotis simus*-like from southern South America

All Argentinian specimens of this taxon have been collected in flooded areas and wetland biomes of the Humid Chaco ecoregion (following Olson et al. 2001). Specimens from Corrientes are from “Los Esteros del Iberá”, one of the largest South America’s freshwater wetlands (Cózar et al. 2005). This region is similar to the Brazilian Pantanal, the region where we found other *simus*-like specimens. The holotype of *M. guaycuru* was collected in the Cerrado ecoregion (Olson et al. 2001), in an area geographically close to a Cerrado–Pantanal ecotone, near to the border of Paraguay (Fig. 1, loc. 5).

We envision two more realistic scenarios for this puzzle. Assuming that those specimens from mid-western Brazil (Pantanal and Cerrado) are conspecific with *M. simus* from the Amazon Basin, and those from Central Paraguay and north-eastern Argentina are *M. simus* as well; Amazon basin populations of *M. simus* can be connected with southernmost populations throughout the Brazilian savannah (Cerrado [Fig. 1, loc. 5]) and wetlands (Pantanal [Fig. 1, loc. 6, 7). However, a previous distribution modelling did not retrieve good support for this corridor (see Moratelli et al. 2011b). In another scenario, *M. simus* might be restricted to the Amazon basin, and those from mid-western Brazil and north-eastern Argentina may represent a third species morphologically closer to *M. simus* than to *M. midastactus*. Populations of this third species can be connected by central Paraguayan populations throughout the Humid Chaco. If future research confirms this second hypothesis, *Myotis guaycuru* Proença, 1943 is the name available for this taxon.

## Supplementary Material

Supplementary material 1 Material examined Data type: Specimens examined and geographical coordinates of their localities
Brief description: Vouchers of *Myotis midastactus*, *M. simus*, and *M. simus*-like and the geographical coordinates of their localities are listed in the supplementary material. They are housed in the following institutions: American Museum of Natural History ([AMNH], New York, USA); National Museum of Natural History ([USNM], Washington DC, USA); Museu de Zoologia da Universidade de São Paulo ([MZUSP], São Paulo, Brazil); Universidade Federal Rural do Rio de Janeiro ([ALP], Seropédica, Brazil); Colección de Mamíferos de la Fundación Miguel Lillo ([CML], San Miguel de Tucumán, Argentina); Museo Argentino de Ciencias Naturales “Bernardino Rivadavia” ([MACN], Ciudad Autónoma de Buenos Aires, Argentina); Colección Teriológica del Instituto de Biología de la Universidad de Antioquia ([CTUA], Medellín, Colombia); and Colección de Mamíferos del Instituto de Ciencias Naturales ([ICN], Bogotá DC, Colombia).
File: oo_49979.pdf Moratelli R, Idárraga L, Wilson DE

## Figures and Tables

**Table 1.** Selected measurements (mm) and body mass (g) of *Myotis midastactus* from Bolivia (type series) and Paraguay (MVZ 144481–144484), *M. simus* from the Amazon Basin (Brazil, Colombia, Ecuador, and Peru), *M. simus*-like from Argentina, and the holotype of *M. guaycuru* (ALP 9277; female) from mid-western Brazil. Samples include adults only, with males and females combined. See Material and methods for variable abbreviations.

	***M. midastactus***		***M. simus***	***M. simus*-like**	***M. guaycuru***
	**Bolivia**	**Paraguay**	**Amazon Basin**	**Argentina**	**Brazil**
	Mean	Mean	Mean	Mean	Holotype
	(Min–Max) N	(Min-Max) N	(Min-Max) N	(Min-Max) N	ALP 9277
TL	90	97	85	86	75
	(86–93) 10	(96–100) 4	(83–87) 2	(83–93) 4	–
Tail	39	39	33	36	33
	(36–40) 10	(37–41) 4	(33–33) 2	(30–40) 4	–
HF	9	10	8	9	7*
	(8–10) 10	(9–11) 4	(7–9) 3	(8–9) 4	–
Ear	13	14	12	13	12
	(12–13) 10	(13–15) 3	(11–13) 3	(12–13) 4	–
BM	10	9	7	9	–
	(6–11) 15	(8–10) 4	(5–8) 2	–	–
FA	39.3	38.5	37.8	39.0	38.1
	(38.2–40.7) 17	(38.2–39.1) 4	(35.5–39.7) 29	(38.8–39.1) 4	–
3ML	36.1	35.2	​34.7	35.8	35.6
	(34.5–37.9) 17	(34.8–35.6) 4	(32.7–36.9) 35	(34.9–35.3) 4	–
LDH	5.0	5.0	4.0	4.0	–
	(3.0–6.0) 17	(4.0–5.0) 4	(3.1–5.3) 29	(4.0–4.0) 3	–
LVH	4.0	4.0	3.4	3.0	–
	(3.0–5.0) 17	(3.0–5.0) 4	(2.8–4.2) 29	(2.0–4.0) 3	–
GLS	14.6	14.3	14.1	13.8	13.7
	(13.9–15.1) 26	(14.0–14.5) 4	(13.6–14.8) 43	–	–
CCL	13	12.8	12.4	11.4	12.2
	(12.4–13.4) 25	(12.5–13.0) 4	(11.9–13.0) 40	–	–
CBL	13.6	13.5	13.0	13.4	12.9
	(13.2–14.0) 25	(13.3–13.7) 4	(12.5–13.5) 40	–	–
CIL	13.9	13.7	13.3	13.5	13
	(13.3–14.2) 25	(13.4–13.9) 4	(12.7–13.9) 41	–	–
BAL	12.4	12.2	11.8	11.3	11.6
	(11.8–12.7) 25	(12.0–12.6) 4	(11.2–12.4) 40	–	–
ZB	9.9	–	9.1	8.9	8.8
	(9.6–10.2) 15	–	(8.2–9.5) 11	–	–
MAB	8.2	8.0	7.5	7.8	7.4
	(8.0–8.5) 26	(7.8–8.1) 4	(6.9–8.1) 33	–	–
BCB	7.4	7.1	6.9	7.0	7.0
	(7.1–7.7) 26	(7.0–7.2) 4	(6.6–7.3) 39	–	–
IOB	5.0	4.8	4.8	4.7	4.9
	(4.8–5.3) 26	(4.8–5.0) 4	(4.5–5.0) 44	–	–
POB	4.0	4.0	3.8	3.8	3.9
	(3.8–4.3) 26	(4.0–4.0) 4	(3.6–4.0) 44	–	–
BAC	4.2	4.0	4.1	3.7	3.9
	(4.0–4.5) 26	(3.9–4.1) 4	(3.7–4.5) 42	–	–
BAM	6.1	5.8	5.7	5.5	5.5
	(4.1–6.4) 26	(5.7–6.0) 4	(5.3–6.0) 44	–	–
MTL	5.4	5.3	5.1	5.0	5.0
	(4.2–5.6) 26	(5.3–5.4) 4	(4.9–5.3) 43	–	–
M1–3	3.2	3.1	3.0	3.0	3.0
	(4.3–3.3) 26	(3.0–3.2) 4	(2.7–3.2) 44	–	–
MAL	10.7	10.5	10.1	10.2	10.0
	(4.4–11.0) 23	(10.3–10.7) 4	(9.5–10.5) 15	–	
MAN	5.8	5.5	5.4	5.4	–
	(4.5–6.0) 24	(5.5–5.7) 4	(5.2–5.8) 43	–	–
* Measured without claw					
