# Supplementary material for: Range extension of Myotis midastactus​ (Chiroptera, Vespertilionidae) to Paraguay
Source: Biodivers Data J. 2015 Aug 18;(3):e5708. doi: 10.3897/BDJ.3.e5708 (PMC4568408; doi:10.3897/BDJ.3.e5708)
Supplement: Supplementary material 1 — Material examined [file biodiversity_data_journal-3-e5708-s001.pdf]

SUPPLEMENTARY FILE OF Range extension of *Myotis midastatctus* to Paraguay BY MORATELLI, IDÁRRAGA AND WILSON

Specimens examined and geographical coordinates of their localities

Vouchers examined are housed in the following institutions: American Museum of Natural History ([AMNH], New York, USA); National Museum of Natural History ([USNM], Washington DC, USA); Museu de Zoologia da Universidade de São Paulo ([MZUSP], São Paulo, Brazil); Universidade Federal Rural do Rio de Janeiro ([ALP], Seropédica, Brazil); Colección de Mamíferos de la Fundación Miguel Lillo ([CML], San Miguel de Tucumán, Argentina); Museo Argentino de Ciencias Naturales "Bernardino Rivadavia" ([MACN], Ciudad Autónoma de Buenos Aires, Argentina); Colección Teriológica del Instituto de Biología de la Universidad de Antioquia ([CTUA], Medellín, Colombia); and Colección de Mamíferos del Instituto de Ciencias Naturales ([ICN], Bogotá DC, Colombia). Localities are arranged by species and alphabetically by country and by major political unit (department/state, province/county). For locality numbers see Figure 1. Coordinates were obtained from different sources, including: skin tags, Gardner's (2008) Gazetteer of Marginal Localities, Google Earth Program, cartographic directory (dices.net, mapcarta.com), Peru's digital nomenclator of the "Instituto Geográfico Nacional" (http://www.ign.gob.pe), Anderson (1997), and L. Emmons (pers. comm.).

Myotis midastactus (N = 33; 30 ♀♀, 3 ♂♂): BOLIVIA: Beni, Cercado, Rio Mamoré, about 23 km W of San Javier (loc. 1; 14°35′45.60″ S, 65°00′0.36″ W, 158 m [AMNH 211155, 211156—holotype, 211167–211176, 211178–211184, 211190, 211192–211198, CML 2261]); Santa Cruz, El Refugio de Huanchaca (loc. 4; 14°45′59.00″ S, 61°2′5.00″ W, 179 m [USNM 584502]). PARAGUAY: Presidente Hayes, Villa Hayes 230 km by road NW (loc. 19; ≈ 23°33′48.84″ S, 58°45′31.94″ W, 100 m [MVZ 144481–144484]). The following material from the vicinities of the type locality was not examined but we assumed they correspond to *M. midastactus*. BOLIVIA (Anderson 1997): Beni, near Espíritu (loc. 3; ≈ 14°13′ S, 66°40′ W); Curiraba River (loc. 2; 14°50′ S, 66°23′ W).

Myotis simus (N = 79; 44 ♀♀ [including 12 sub-adults], 31 ♂♂ [including 10 sub-adults], 4 sex undetermined): BRAZIL: Amazonas, Borba (loc. 10; 04°23′36.12″ S, 59°35′30.46″ W, 29 m [AMNH 91886–91892, 94224, 94225, 94227, 94230–94234]); Amazonas, Itacoatiara (loc. 9; 03°8′12″ S, 58°26′0.02″ W, 20 m [MZUSP 3472]); Amazonas, Manaus, Rio Negro, Igarapé Cacau Pereira (loc. 11; 03°08′60.00″ S, 60° 07′0.00″ W, 13 m [AMNH 79534, 91472–91478, 91500]); Amazonas, Parintins (loc. 8; 02°38′0.0″ S, 56°44′0.0″ W, 16 m [AMNH 92983, 93489–93497, 93922–93925]); Amazonas, Rio Juruá (loc. 12; 5°38′44.28″ S, 67°43′40.23″ W, 95 m [MZUSP 638, 1074]); unknown locality (MZUSP 1062). COLOMBIA: Amazonas, Leticia, Parque Nacional Natural Amacayacu (loc. 25; 03°25′51.21″ S, 70° 08′14.79″ W, 148 m [CTUA 11161]); Amazonas, unknown locality (TTU 9073, 9076–9078); Caquetá, Florencia, Vereda Santo Domingo, Granja Experimental Santo Domingo, Universidad de la Amazonía (loc. 26; 01°35′8.33″ N, 75°38′31.93″ W, 285 m [ICN 16917]). PERU: Loreto, Maynas, Las Amazonas, Apayacu, Amazon river (loc. 27; 03°29′7.00″ S, 72°11′6.00″ W, 84 m [AMNH 74378–74381]); Loreto, Maynas, Amazonas, San José de Orosa (loc. 14; 03°26′47.81″ S, 72°04′26.43″ W, 85 m [AMNH 74105, 74109, 74110]); Loreto, Maynas, Napo, Curaray river mouth (loc. 15; 02°21′52.03″ S, 74°05′38.24″ W, 135 m [AMNH 71483, 71485–71488, 71490–71494]); Loreto,

Ucayali, Sarayacu, Ucayali river (loc. 16; 06°43′58.82″ S, 75°06′0.29″ W, 129 m [AMNH 76240–76249, 76252, 76253—topotypes of *M. simus*]); Pasco, Oxapampa, San Juan (loc. 17; 10°29′25.23″ S, 74°49′1.41″ W, 289 m [USNM 364481, 364482]). The following material was not examined but we assumed they correspond to *M. simus*. COLOMBIA (Wilson 2008): Amazonas, Leticia (loc. 13; 04°09′ S, 69°57′ W); PERU (Voss and Emmons 1996): Ucayali, Balta, Cashinaua indian village (loc. 18; 10°08′ S, 71°13′ W).

Myotis simus-like (N = 8; 1 ♀, 7 ♂♂ [including 2 sub-adults]): ARGENTINA: Corrientes, Ituzaingó, Isla Apipé (loc. 24; 27°30′32.62″ S, 56°52′51.42″ W, 60 m [MACN 18033]); Corrientes, Mburucuyá, Parque Nacional Mburucuyá, Estancia Santa Teresa (loc. 28; 28°01′59.88″ S, 58°01′59.88″ W, 69 m [MACN 20914]); Formosa, Pilcomayo, Parque Nacional Río Pilcomayo, Paso Pomelo (loc. 20; 25°00′52.20″ S, 58°07′9.12″ W, 81 m [CML 4680, MACN 20901]). BRAZIL: Mato Grosso, Taiamã (loc. 6; 16°50′42.40″ S, 57°31′26.22″ W, 107 m [MZUSP 13815]); Mato Grosso, Barão de Melgaço (loc. 7; 16°11′46.28″ S, 55°58′2.71″ W, 138 m [MN 71451, 71458]); Mato Grosso do Sul, Salobra, rio Miranda (loc. 5; 20°11'16.75″ S, 56°30′26.08″ W, 118 m [ALP 9277—holotype of *M. guaycuru*]). The following material was not examined but we assumed they correspond to *M. simus*-like: ARGENTINA (Bárquez et al. 1999): Formosa, Pilcomayo, Estancia Las Mercedes (loc. 21; 25°11′ S, 57°54′ W). PARAGUAY (López-González 2005, Wilson 2008): Central, Luque, 17 km by road E (loc. 22; ≈ 25°16′ S, 57°26′ W); Misiones, Ayolas, Barrio San Antonio (loc. 23; 27°23.42′ S, 56°50.15′ W).

References

Anderson S (1997) Mammals of Bolivia, taxonomy and distribution. Bulletin of the American Museum of Natural History 231: 1–652.

Bárquez RM, Mares MA, Braun JK (1999) The bats of Argentina. Special Publication of Texas Tech University and the Oklahoma Museum of Natural History 42: 1–275.

López-González C (2005) Murciélagos de Paraguay. Biosfera, Sevilla, iv + 300 pp.

Voss RS, Emmons LH (1996) Mammalian diversity in Neotropical lowland rainforest: a preliminary assessment. Bulletin of the American Museum of Natural History 230: 1–115.

Wilson DE (2008) Genus *Myotis* Kaup 1829. In: Gardner A (Ed.) Mammals of South America, volume 1, marsupials, xenarthrans, shrews, and bats. University of Chicago Press, Chicago, xiii + 669.
